# Polymer-Cement Composites Containing Waste Perlite Powder

**DOI:** 10.3390/ma9100839

**Published:** 2016-10-17

**Authors:** Paweł Łukowski

**Affiliations:** Faculty of Civil Engineering, Warsaw University of Technology, al. Armii Ludowej 16, Warsaw 00-637, Poland; P.Lukowski@il.pw.edu.pl

**Keywords:** cement, composite, construction, modification, perlite, polymer, utilization, waste

## Abstract

Polymer-cement composites (PCCs) are materials in which the polymer and mineral binder create an interpenetrating network and co-operate, significantly improving the performance of the material. On the other hand, the need for the utilization of waste materials is a demand of sustainable construction. Various mineral powders, such as fly ash or blast-furnace slag, are successfully used for the production of cement and concrete. This paper deals with the use of perlite powder, which is a burdensome waste from the process of thermal expansion of the raw perlite, as a component of PCCs. The results of the testing of the mechanical properties of the composite and some microscopic observations are presented, indicating that there is a possibility to rationally and efficiently utilize waste perlite powder as a component of the PCC. This would lead to creating a new type of building material that successfully meets the requirements of sustainable construction.

## 1. Introduction

The utilization of the waste materials for the production of building composites is required for sustainable development in construction [1]; results of the Life Cycle Analysis proved the effectiveness of this kind of solutions [2,3]. Such wastes are, for example, fly ash or blast-furnace slag [4,5,6], used over the years in the production of cement and concrete. Recently, the need for utilization of the other waste materials, such as perlite powder, has arisen. The subject of this paper is evaluation of the possibility of use of waste perlite powder as a component of polymer-cement composites (PCCs).

Perlite is a material of volcanic origin. It is thermally treated in order to obtain its expanded form, which has several advantageous properties such as low thermal conductivity and a high ability of absorbing liquids and gases in combination with low density. Expanded perlite is commonly used as a component of lightweight concretes and heat-insulating plasters and mortars as well as various types of thermal and acoustic insulations [7]. However, during the treatment of natural perlite, a large amount (5%–10% of the product) of powder is created. Having fine particles (below 50 µm in diameter) and very low bulk density (usually at the level 100 kg/m^3^), the waste perlite powder is onerous and expensive in storage and is possibly re-processing. The annual world production of expanded perlite is about 1.7 million tons; an average production facility generates annually about 3–8 thousand cubic meters of perlite powder and the estimated volume of the annual production of this waste in Poland is about 20–25 thousand cubic meters [8]. The storage capability of the Polish perlite production facilities is now mostly exhausted. At present, only a very small amount of perlite powder is recycled, mainly by adding it to perlite with a coarser grain size, which leads to a worsening of product quality. Thus, there is a need for finding other ways of the utilization of waste perlite powder.

Many studies have been done on the performance of expanded perlite as a replacement for the part of the aggregate in concrete. These works have covered thermal conductivity and mechanical strength of the lightweight concrete containing perlite [9,10,11] and applying perlite in self-compacting concrete [12,13].

Some information about the use of perlite powder as a partial replacement for cement in ordinary concrete can also be found. Yu et al. have found that natural perlite powder has quite good pozzolanic properties [14]. They have also investigated the influence of perlite admixture on the pore structure in cement paste, finding that it diminishes porosity, decreases pore diameter, and reduces pore surface area [15]. Erdem et al. have proposed using perlite as an addition in blended cements [16]; Erdoğan and Sağlık have found that large amounts of unexpanded perlite worsen the early-age strength of cement mortars, but that this can be overcome by the chemical or thermal activation of the perlite [17]. Bektas et al. have noticed that the admixture of both natural and expanded perlite powder can inhibit the deleterious alkali-aggregate reaction in concrete [18]. Recently, Ramezanianpour et al. [19] and Vosoughi et al. [20] have studied the possibility of partial substitution of cement by expanded perlite powder during concrete production.

PCCs are the materials in which the polymer and mineral binder create an interpenetrating network [21]; the co-operation of these two binders brings the improvement of flexural and tensile strength, adhesion to various substrates, and tightness [22]. The main fields of use of PCCs are repair and protection of concrete structures, industrial floors, and precast building elements. As for now, few authors have considered the possibility of introducing mineral additives as components of polymer concretes or polymer-cement concretes [23], since they have been generally considered as poorly compatible with polymers [24]. Evbuomwan [25] and Gao et al. [26] have confirmed the strengthening effect of microsilica in polymer-cement concrete, while Bonora et al. have studied the influence of fly ash content on the resistance of PCCs to environmental attack [27]. Recently, Sikora et al. have reported an attempt to introduce nanosilica into a PCC [28].

Jaworska et al. recently pointed out the possibility of using waste perlite powder as a PCC component and evaluated the chemical resistance of such composites [29]. In this paper, the influence of perlite powder on the mechanical properties of PCCs is presented, and the utilization of this burdensome waste as a partial substitute for cement in PCCs is discussed.

## 2. Materials and Methods

### 2.1. The Scope of Investigation and Materials Used

The first part of the investigation covered the characterization of waste perlite powder. Chemical composition, bulk density, and granulometry of the perlite powder were determined.

Then, tests were carried out on polymer-cement mortars containing waste perlite powder, with a binder-to-sand ratio equal to 1:3 by mass and a water-to-binder ratio equal to 0.5. The components of the PCCs were as follows:

- Portland cement CEM I 42.5R according to European Standard EN 197-1 “Cement—Part 1: Composition, specifications and conformity criteria for common cements” [30];
- A commercially available polymer modifier—styrene-acrylic co-polymer (SA);
- Waste perlite powder from an expanded perlite production facility;
- CEN standard sand 0–2 mm according to European Standard EN 196-1 “Methods of testing cement—Part 1: Determination of strength” [31];
- Tap water.

The reference material was unmodified, standard Portland cement mortar according to European Standard EN 196-1 (a cement-to-sand ratio equal to 1:3 and water-to-cement ratio equal to 0.5). The polymer-to-cement ratio in polymer-cement mortars was 0.05, 0.10, and 0.20 (by mass). For each level of polymer content, the cement was substituted by 0% to 25% (by mass) of waste perlite powder. The mixture compositions are presented in Table 1. The selected properties of polymer-cement mortars containing waste perlite powder include flexural strength, tensile strength, and compressive strength, which were determined after 28 and 90 days of curing. The activity index of the perlite powder was also determined. The activity index of the waste perlite powder, according to European Standard EN 450-1 [32] “Fly ash for concrete—Part 1: Definition, specifications and conformity criteria”, is the ratio of the compressive strength of standard mortar, prepared with 75% cement and 25% investigated material by mass, to the compressive strength of standard mortar, prepared with 100% cement, when tested at the same age. Additionally, the maximum elongation at tension was determined after 28 and 90 days of curing, and the density of the mortars was determined after 90 days of curing in order to analyze the relation between the density and the strength of the composite.

### 2.2. Preparation of Specimens and Methods of Testing

The tests of flexural strength and compressive strength of the mortars were conducted using prism specimens with dimensions 4 × 4 × 16 cm^3^, prepared according to European Standard EN 196-1. The same specimens were used to determine the density of the mortars. For tensile strength and elongation testing, the “eight-shaped” specimens of the mortars were prepared according to Polish Standard PN-B-04500 [33] “Mortars–Testing of mechanical properties”.

The mixed regime of curing, as it is generally recommended for PCCs [34], was applied for the polymer-cement mortars: the specimens were tightly covered with plastic sheet for 1 day, demolded, immersed in water at a temperature of 20–22 °C for 5 days (for promotion of cement hydration), and then stored in a laboratory air-dry environment at a temperature of 20–22 °C and with 60% ± 5% relative humidity (for promotion of polymer hardening) until testing. The reference cement mortars (without the polymer) were cured under water with a temperature of 20–22 °C, according to European Standard EN 196-1.

The chemical composition of tested perlite powder was determined via wavelength dispersive X-ray fluorescence (WDXRF). The bulk density of the powder was determined via the gravimetric method, and the granulometry of the powder was determined by the use of the laser scattering method.

The flexural strength, tensile strength, elongation, and density of the mortars were determined according to Polish Standard PN-B-04500, while compressive strength was determined according to European Standard EN 12190 [35] “Products and systems for the protection and repair of concrete structures–Test methods–Determination of compressive strength of repair mortar”. A computer-controlled testing machine was used. A laser optical microscope and a high-resolution electron scanning microscope were employed for obtaining microscopic images.

## 3. Results and Discussion

### 3.1. Characteristics of the Waste Perlite Powder

The chemical composition of the waste perlite powder is presented in Table 2. For comparison, the chemical composition of the Portland cement used in testing is also presented. The two materials differ significantly in the content of the main constituents. The waste perlite is composed mainly of SiO_2_ and Al_2_O_3_, while the dominant constituent of cement is CaO.

**Figure 1:**
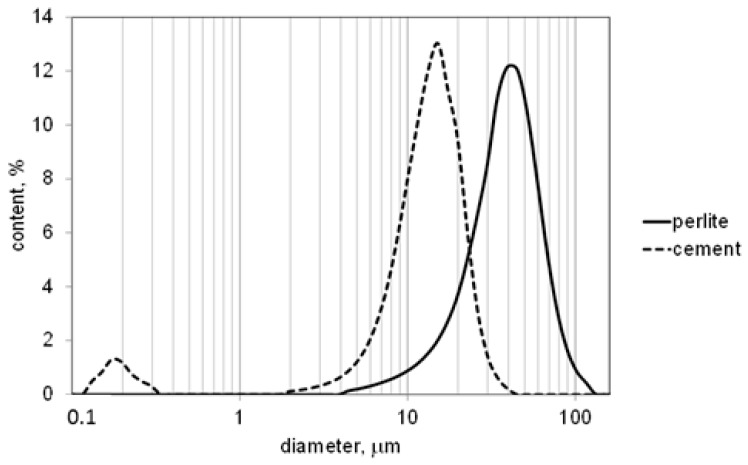
Grain size distribution of the waste perlite powder.

Grain size distribution of the waste perlite powder is presented in Figure 1 together with the grain size distribution of the Portland cement used in testing. The mean diameter of the grains of the waste perlite was 37 μm, and 90% of the grains had a diameter (D_90_) below 59 μm. The mean diameter of the grains of the cement was 13 μm, and 90% of the grains had a diameter (D_90_) below 22 μm.

**Figure 2:**
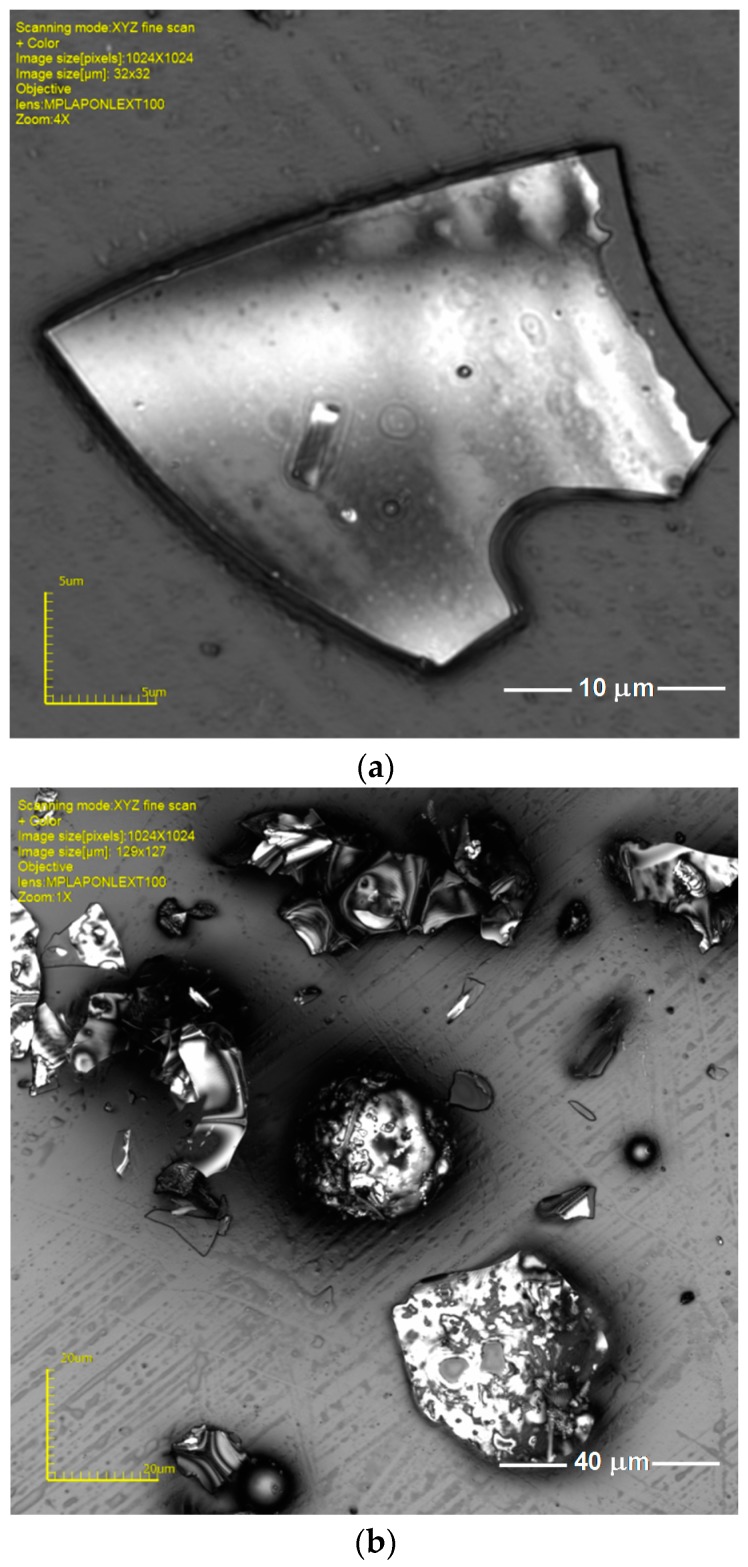
Typical shapes of the waste perlite powder grains; images from a laser optical microscope. (**a**) Single flat particle; (**b**) particles of irregular shapes.

The microscopic observation showed that the perlite powder particles are in the shape of plates, flakes, fragments of spheres, and other irregular forms, which together create irregular particles (Figure 2). Such morphology of the waste perlite powder results negatively in two ways. Firstly, the developed amorphous structures may deteriorate the workability of the concrete or mortar mix. This can lead to a less uniform and therefore worse compacted structure of hardened material and, consequently, to the deteriorated mechanical performance of the composite. Secondly, the material itself characterizes with a very low value of bulk density, which has been determined to be equal to 92 kg/m^3^, so the waste requires enormous space for storing. Thus, the application of even a small (by weight) amount of perlite powder in the composite is the utilization of a large volume of expensively stored waste.

### 3.2. Test Results of the Polymer-Cement Mortars Containing Waste Perlite Powder

**Figure 3:**
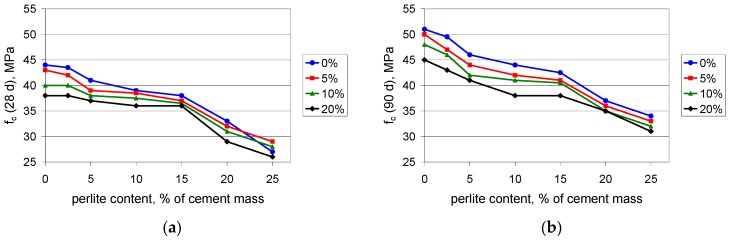
Compressive strength of mortars with polymer content values of 0%, 5%, 10%, and 20% of cement mass vs. waste perlite content: (**a**) after 28 days; (**b**) after 90 days of curing.

**Figure 4:**
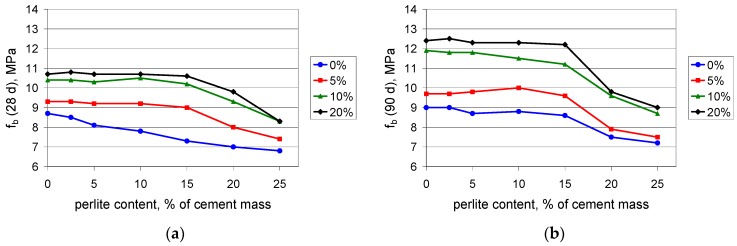
Flexural strength of mortars with polymer content values of 0%, 5%, 10%, and 20% of cement mass vs. waste perlite content: (**a**) after 28 days; (**b**) after 90 days of curing.

**Figure 5:**
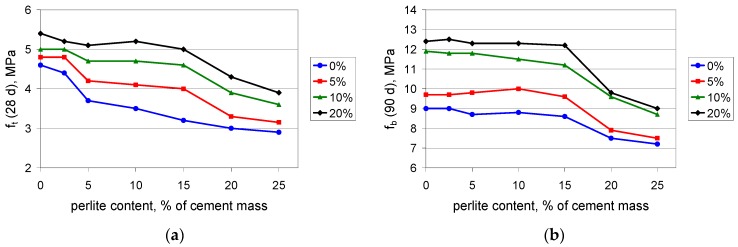
Tensile strength of mortars with polymer content values of 0%, 5%, 10%, and 20% of cement mass vs. waste perlite content: (**a**) after 28 days; (**b**) after 90 days of curing.

**Figure 6:**
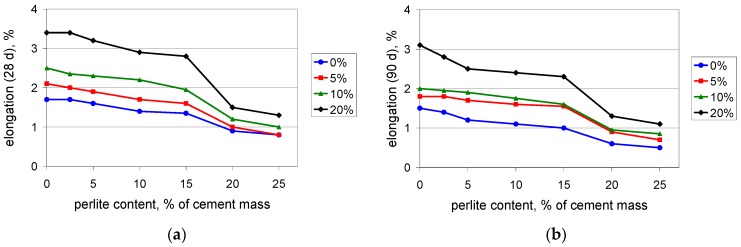
Maximum elongation at tension of mortars with polymer content values of 0%, 5%, 10%, and 20% of cement mass vs. waste perlite content: (**a**) after 28 days; (**b**) after 90 days of curing.

**Figure 7:**
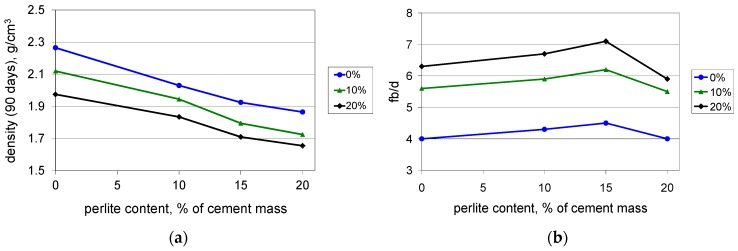
Density (**a**) and flexural strength to density ratio (**b**) of the mortars with polymer content values of 0%, 10%, and 20% of cement mass vs. waste perlite content after 90 days of curing.

The investigation of PCC mortars with waste perlite powder covered a determination of compressive, flexural, and tensile strength as well as maximum elongation at tension, after 28 and 90 days of curing. Because the light weight is an advantage of the PCCs containing perlite powder, the density of the mortars was determined. The test results are presented in Figure 3, Figure 4, Figure 5, Figure 6 and Figure 7.

The compressive strength of the tested composites decreases with an increasing amount of polymer, which is not surprising since polymers delay the hydration of cement [36]. The introduction of waste perlite powder at a content value of up to 15% of the cement mass causes a slight decrease in the compressive strength of PCC mortars. With such an amount of waste, the 28-day compressive strength (Figure 3a) is reduced by 5%–10% as compared with the mortar without perlite. A larger amount of waste leads to a sharper decrease in compressive strength; when the content value of the perlite is 20%, the reduction in compressive strength significantly exceeds 20%. The 90-day compressive strength (Figure 3b) is reduced by about 15% when the content value of waste perlite is 15%, while at a content value of 20% the reduction reaches almost 30%.

A determination of the compressive strength of the mortars without polymers allows us to calculate the activity index of waste perlite powder used in the investigation (Table 3).

The obtained values of the activity index of waste perlite powder are substantially lower than those required for fly ashes when used as mineral additives to concrete. According to EN 450-1, the activity index of the fly ash should be at least 75% after 28 days of curing and at least 85% after 90 days. Nevertheless, the results show that the waste perlite powder has some pozzolanic ability, which has been confirmed by the results of strength determinations.

Flexural and tensile strength increases when polymer is added to the composite. This is in perfect agreement with the expectations, since the polymer modifier is expected to improve these properties [37]. The test results confirm the observation from the compressive strength determination: when the content value of the perlite waste is up to 15%, the performance of the composite is rather moderate. At 15%, the waste perlite causes a reduction in the 28-day flexural strength (Figure 4a) by no more than 3%, except in the case of 20% of polymer, where the reduction reaches 15%. A larger amount of powder worsens the mechanical properties. When the content value of the perlite waste is 20%, the reduction of the 28-day flexural strength is 15%–20%. The 90-day flexural strength (Figure 4b) is reduced by no more than 5% when the content value of the waste perlite is 15%, while at a content value of 20% the reduction exceeds 20%. A similar situation can be observed for the tensile strength (Figure 5). The same conclusion can be drawn from the results of the testing of the maximum elongation at tension. The elongation after 90 days (Figure 6b) is somewhat lower than that after 28 days (Figure 6a), which shows that the composite containing the waste perlite powder becomes more brittle with time.

The density of the investigated PCCs decreases with increasing content values of polymer and waste perlite powder (Figure 7a). The flexural strength to density ratio shows a favorable maximum when the content value of the waste perlite is approximately 15% (Figure 7b).

An important advantage of perlite powder is its ability to suppress the deleterious expansion induced by a alkali–silica reaction, confirmed in experimental investigations [18]; it is assumed that the effectiveness of expanded perlite is based both on pozzolanic activity and on the space provided for the gel accommodation due to its porous nature. These assumptions can be fully adopted to the waste perlite powder due to its pozzolanic ability and the morphology of its particles, analyzed in this paper.

### 3.3. SEM Observations

**Figure 8:**
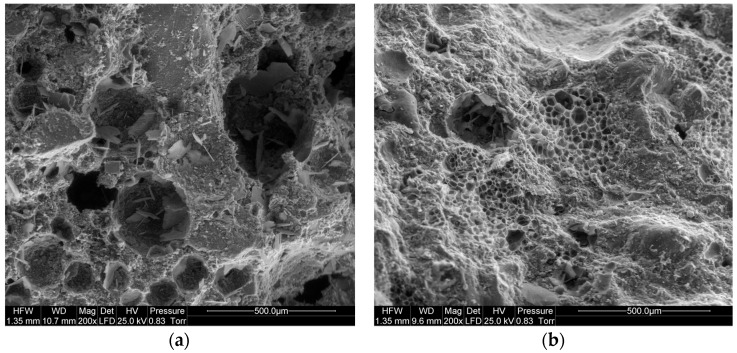
Microstructure of polymer-cement mortar containing waste perlite powder; content values of additions (in relation to cement mass): (**a**) perlite 10%, polymer 5%; (**b**) perlite 10%, polymer 15%.

**Figure 9:**
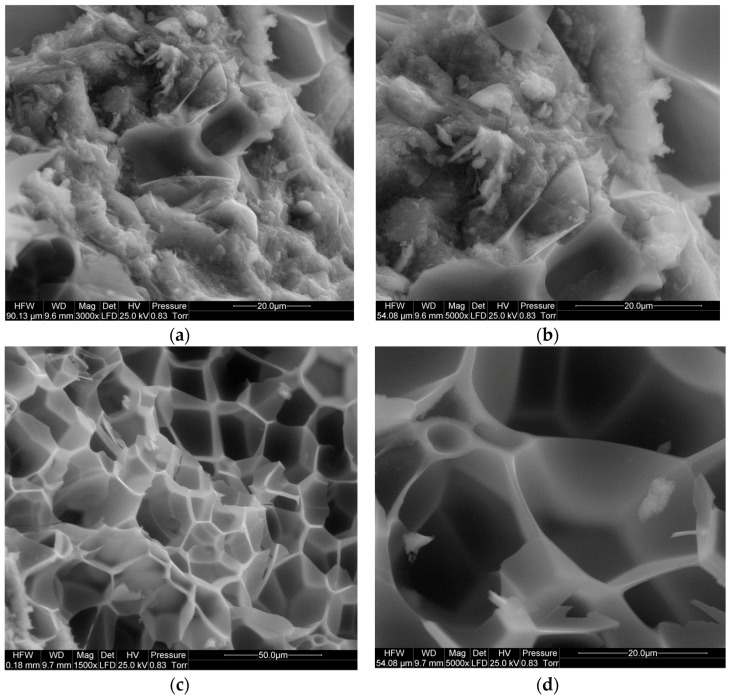
Microstructure of polymer-cement paste containing waste perlite powder; content values of the additions (in relation to cement mass): perlite 15%, polymer 20%. (**a**) polymer-cement interpenetrating network; (**b**) closer look to polymer-cement network; (**c**) polymer network; (**d**) detail of polymer network with embedded particles of perlite powder.

The subject of the scanning microscope observations was the microstructure of the PCCs containing waste perlite powder as the partial replacement for cement. The microstructure of the PCCs containing 10% waste perlite powder (in relation to cement mass) is presented in Figure 8. At a lower amount of polymer, the microstructure of the composite is less uniform. The addition of a larger amount of the polymer modifier makes it more homogenous, with a uniformly dispersed polymer network. The microstructure of the polymer-cement paste is presented in Figure 9; the perlite powder particles of irregular shape are embedded into the polymer film. This can cause the additional delaying in the pozzolanic action of perlite; however, on the other hand, more homogenous structure compensates, to some extent, for this negative effect.

## 4. Summary and Conclusions

The need for utilization of the waste materials as the components of the building materials is a demand of sustainable construction. Waste mineral powders, such as fly ash or blast-furnace slag, are successfully used for the production of cement and concrete. Perlite powder is a burdensome waste substance from the process of thermal expansion of the raw perlite. Due to its very low bulk density, the material is difficult for storage. Because of some pozzolanic ability, it was considered a partial substitution for cement in building materials. In this paper, a possibility of the utilization of waste perlite powder for the manufacture of PCCs is presented. The test results show that

- replacing up to 15% of the cement (by mass) with perlite powder does not cause significant deterioration in mechanical performance of the PCC;
- replacing part of the cement with perlite powder decreases the density of the composite—the favorably highest ratio of strength to density can be observed at 15% content value of perlite powder, irrespective of polymer content;
- the presence of a polymer modifier makes the microstructure of the composite more homogenous, which can compensate for the delay of pozzolanic action of the mineral addition and foster a rational and efficient utilization of the waste;
- further investigation should be focused on the development of suitable technology of the manufacture of polymer-cement products containing waste perlite powder.

## Figures and Tables

**Table 1:** Mixture compositions (in g).

Series	Mix No.	Cement	Water	Sand	Polymer	Waste Perlite
P0	0-1	450	225	1350	0	0
	0-2	438.75	225	1350	0	11.25
	0-3	427.5	225	1350	0	22.5
	0-4	405	225	1350	0	45
	0-5	382.5	225	1350	0	67.5
	0-6	360	225	1350	0	90
	0-7	337.5	225	1350	0	112.5
P5	5-1	450	225	1350	5	0
	5-2	438.75	225	1350	5	11.25
	5-3	427.5	225	1350	5	22.5
	5-4	405	225	1350	5	45
	5-5	382.5	225	1350	5	67.5
	5-6	360	225	1350	5	90
	5-7	337.5	225	1350	5	112.5
P10	10-1	450	225	1350	10	0
	10-2	438.75	225	1350	10	11.25
	10-3	427.5	225	1350	10	22.5
	10-4	405	225	1350	10	45
	10-5	382.5	225	1350	10	67.5
	10-6	360	225	1350	10	90
	10-7	337.5	225	1350	10	112.5
P20	20-1	450	225	1350	20	0
	20-2	438.75	225	1350	20	11.25
	20-3	427.5	225	1350	20	22.5
	20-4	405	225	1350	20	45
	20-5	382.5	225	1350	20	67.5
	20-6	360	225	1350	20	90
	20-7	337.5	225	1350	20	112.5

**Table 2:** Chemical composition of the waste perlite powder and cement CEM I 42.5R used in testing.

Component	Content in Perlite, Mass %	Content in Cement, Mass %
SiO_2_	73.74	22.18
Al_2_O_3_	13.12	5.98
Fe_2_O_3_	1.25	2.82
CaO	1.23	61.13
MgO	0.03	1.12
Na_2_O	3.42	0.43
K_2_O	4.20	0.22
TiO_2_	0.08	<0.01
MnO	0.02	<0.01
P_2_O_5_	0.02	<0.01
SO_3_	<0.01	2.69
Cl	0.07	0.04
F	0.05	<0.01
Loss on ignition	2.70	2.61

**Table 3:** Activity index of the waste perlite powder.

Time of Testing	28 Days	90 Days
Average compressive strength of the mortar with the binder containing 75% of cement and 25% of the waste perlite powder	73.74	34 MPa
Average compressive strength of the mortar with the binder containing 100% of cement	44 MPa	51 MPa
Activity index of waste perlite powder	61%	67%
